# Preparation, Characterization of Granulated Sulfur Fertilizers and Their Effects on a Sandy Soils

**DOI:** 10.3390/ma15020612

**Published:** 2022-01-14

**Authors:** Aneta Lisowska, Barbara Filipek-Mazur, Józef Sołtys, Marcin Niemiec, Olga Gorczyca, Dominika Bar-Michalczyk, Monika Komorowska, Zofia Gródek-Szostak, Anna Szeląg-Sikora, Jakub Sikora, Maciej Kuboń

**Affiliations:** 1 Institute of Technology and Life Sciences—National Research Institute, Falenty, 3 Hrabska Av., 05-090 Raszyn, Poland; d.michalczyk@itp.edu.pl; 2 Department of Agricultural and Environmental Chemistry, University of Agriculture in Krakow, 21 Mickiewicza Av., 31-120 Krakow, Poland; barbara.filipek-mazur@urk.edu.pl (B.F.-M.); marcin.niemiec@urk.edu.pl (M.N.); olga.gorczyca@urk.edu.pl (O.G.); monika.komorowska@urk.edu.pl (M.K.); 3 Intermark, Przedsiębiorstwo Techniczno-Handlowe, Św. Marka 9/7, 44-113 Gliwice, Poland; js@intermark.pl; 4 Department of Economics and Enterprise Organization, Cracow University of Economics, 31-510 Krakow, Poland; grodekz@uek.krakow.pl; 5 Faculty of Production and Power Engineering, University of Agriculture in Krakow, 30-149 Krakow, Poland; anna.szelag-sikora@urk.edu.pl (A.S.-S.); jakub.sikora@urk.edu.pl (J.S.); maciej.kubon@urk.edu.pl (M.K.); 6 Institute of Management and Production Engineering, Cavalry Captain Witold Pilecki State University of Małopolska in Oświęcim, Maksymiliana Kolbego 8, 32-600 Oswiecim, Poland; 7 Eastern European State College of Higher Education in Przemysl, Ksiazat Lubomirskich 6, 37-700 Przemysl, Poland

**Keywords:** waste sulfur, elemental sulfur, halloysite, humic acids, fertilizer

## Abstract

There is a potential for using sulfur waste in agriculture. The main objective of this study was to design a granular fertilizer based on waste elemental sulfur. Humic acids and halloysite were used to improve the properties and their influence on soil properties. This is the first report on the use of proposed materials for fertilizer production. The following granular fertilizers were prepared (the percentage share of component weight is given in brackets): fertilizer A (waste sulfur (95%) + halloysite (5%)), fertilizer B (waste sulfur (81%) + halloysite (5%) + humic acids (14%)), fertilizer C (waste sulfur (50%) + halloysite (50%)) and fertilizer D (waste sulfur (46%) + halloysite (46%) + humic acids (8%)). Basic properties of the obtained granulates were determined. Furthermore, the effect of the addition of the prepared fertilizers on soil pH, electrolytic conductivity, and sulfate content was examined in a 90-day incubation experiment. Enrichment with humic acids and the higher amount of halloysite increased the fertilizer properties (especially the share of larger granules and bulk density). In addition, it stabilized soil pH and increased the sulfur content (extracted with 0.01 mol·L^−1^ CaCl_2_ and Mehlich 3) in the soil.

## 1. Introduction

Plant nutrition is an essential issue related to cropping systems, environmental sustainability, and human health. Due to the growing global population, agricultural production must also increase to provide humanity with food in appropriate quantity and quality [1]. It has been assessed that around 60% of global agricultural soils are characterized by growth-limiting factors [2]. Significant issues identified as a worldwide problem reducing agricultural productivity include: soil acidification, sulfur deficiency, and organic matter loss. Acid soils take up 30–40% of arable land, and this area has been growing in recent years. Soil acidification shapes the bio-availability of major nutrients, such as N, P, S, and basic cations. It also determines the mobility of heavy metals; and shapes the microbiological activity, regulating, for instance, the rate of organic matter mineralization [3,4]. Sulfur deficiency has been increasingly noted over the last 50 years worldwide [5,6,7,8,9]. It is also noteworthy that in 2015, 92% of Polish arable soils had insufficient sulfate content to meet the plant needs [9,10]. As mentioned above, organic matter loss is another problem. A decreasing stock of soil organic carbon has been observed, both at the global and regional scale. It has been estimated that carbon inputs must increase by 29% to maintain the present organic carbon stock in croplands [11].

To maintain soil fertility and provide sufficient nutrients to crops, inorganic and organic fertilizers are applied. Properly prepared waste materials may be a valuable alternative to conventional fertilization [12,13]. Recovery of nutrients from waste allows element reintegration into the food chain [14]. This corresponds with the idea of a circular economy and leads to a reduction in the consumption of resources necessary for the production of conventional fertilizers [6,8,11,15,16,17,18,19].

**Figure 1:**
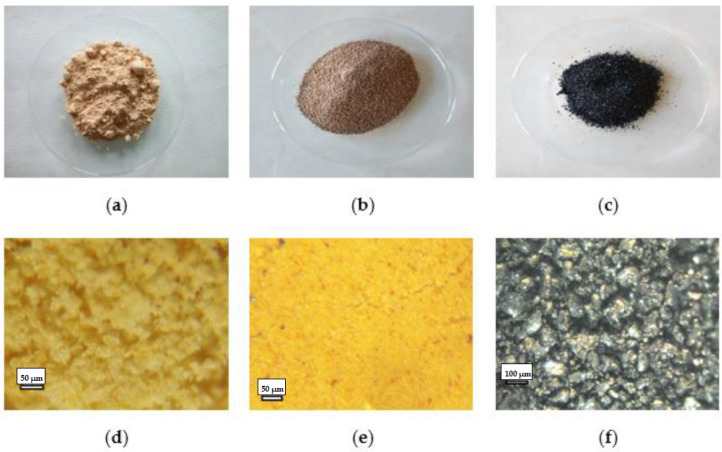
Macroscopic view of dried waste sulfur (**a**), halloysite (**b**) and humic extract (**c**). Optical microscope view of dried waste sulfur (**d**), halloysite (**e**) and humic extract (**f**).

Elemental sulfur (Figure 1a,d) is produced predominantly by recovery from the oil and gas industry. The low price of this material has recently stimulated its wider use [20]. There are various methods of biogas desulfurization. Solid sorbents, biological and sorption methods are used. The methods, based on iron chelating agents under slightly alkaline conditions, include SulFerox^®^ (Royal Dutch Shell plc, Hague, The Netherlands) and Lo-Cat^®^ (Merichem Company, Houston, TX, USA) [21,22]. In Poland, Biosulfex^®^ (PROMIS COMPANY, Warsaw, Poland) is the most commonly used method of desulfurization of gas obtained from sewage sludge digestion. It is based on the use of solutions of iron (III) complexes with organic ligands. Application of this method leads to the elimination of 98% of hydrogen sulfide contained in gas [23,24]. Sulfur is sedimented in the form of pulp; the material contains 80–90% elemental sulfur. Produced sulfur is easily recoverable from the slurry and may constitute a substrate for the production of fertilizers and pesticides. Reuse of this material can be an alternative to soil fertilization with conventional fertilizers and a way to reduce the increasing quantity of waste. However, due to its properties (sludgy form and hydrophobicity), waste sulfur is a difficult material to process.

**Figure 2:**
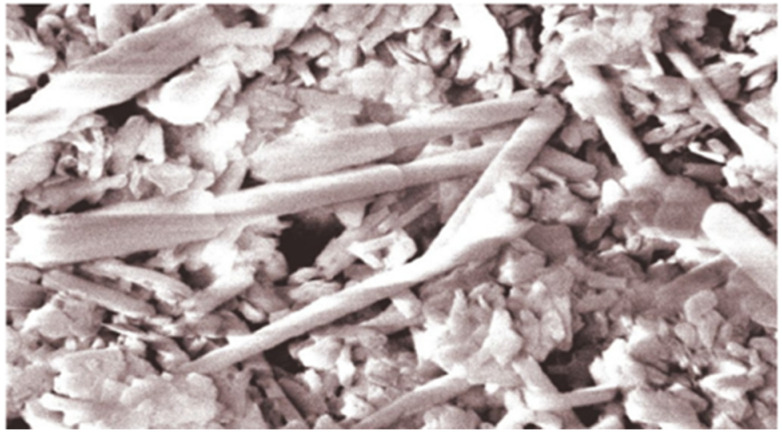
The porous structure of raw halloysite from Dunino deposit in the scanning electron microscope view.

Halloysite (Figure 1b,e and Figure 2), with the empirical formula Al_2_Si_2_O_5_(OH)_4_, chemically related to kaolin, is a naturally occurring aluminosilicate clay mineral. It is a volcanic-derived mineral, formed during basalt weathering. Halloysite has a sorption capacity for neutral molecules and ions. It contains both positively and negatively charged surfaces, and so it is capable of absorbing cations and anions [25]. It has been found that halloysite is effective in reducing heavy metal availability in soil and increasing its pH [26]. Halloysite Dunino retains nutrients and water for a long time and can be used for the production of slow-release fertilizers [27,28]. This feature is in line with the proposal to reduce fertilizer use and nutrient losses which was provided by the European Commission [29,30].

Due to high biocompatibility and low cytotoxicity, halloysite can be used safely in various fields [31]. The “Dunino” open pit mine, located in southwestern Poland, is currently one of three (next to the mine in the USA and New Zealand) that are in operation in the world, with one of the largest deposits of homogeneous halloysite. It is estimated that the deposits range from 10 to 12 million tons. Polish deposits of halloysite are characterized by a unique spatial platelet-tubular structure. It contains mainly halloysite nanotubes and nanoplates [25,32]. The porous structure of raw halloysite from Dunino deposit is shown on Figure 2. In the past, halloysite has been used for various purposes (as: a filler, an adsorbent for pollutant removal from water and process gases, a support for catalytic purposes, a drug carrier) [33], based on various sources. On the other hand, there is a lack of information about its use as soil amendment or fertilizer component.

The main aim of this study was to design a fertilizer based on waste elemental sulfur that would possess adequate physical properties. To achieve that effect, waste sulfur pulp was mixed with specific amounts of humic acids and/or halloysite and granulated. The basic properties of the obtained granulates were determined. Moreover, to evaluate the usefulness of the products, the effect of adding the prepared fertilizers on selected soil properties was examined in an incubation experiment. Based on our knowledge, this is the first report on the use of proposed materials for fertilizer production.

## 2. Materials and Methods

### 2.1. Preparation of Granular Sulfur Fertilizers

To prepare the mixtures from which samples of fertilizer granulates were made, waste sulfur (Figure 1a), halloysite (Figure 1b) and humic extract (Figure 1c) were used. The percent share of individual components mass of granulates is given in brackets:

- waste sulfur (95%) + halloysite (5%);
- waste sulfur (81%) + halloysite (5%) + humic acids (14%);
- waste sulfur (50%) + halloysite (50%);
- waste sulfur (46%) + halloysite (46%) + humic acids (8%).

Granules were prepared according to the procedure adapted to the physical and chemical properties of the raw components, the pressureless mixing method under normal conditions, was used. Materials with natural moisture content were used. In addition, water was used as a binder. The amount of halloysite was adjusted to the conditions of the granulation procedure (a 5% addition of halloysite as a dose sufficient to obtain waste sulfur granules—fertilizers A and B) or increased, in order to assess the influence of halloysite addition on soil properties (fertilizers C and D). Once prepared, the mixtures were dried and crushed. A small mass of the mixtures prevents the use of granulation equipment. That is why crushing was undertaken manually, obtaining granulates with particle size of 0–3 mm.

The waste sulfur used in this study was obtained from a facility located in central Poland. This material is generated as a by-product during desulfurization of biogas with the Biosulfex^®^ method. Waste sulfur had a fine-grained structure (Figure 1d) and contained 32.3% d.m. from which 86.4% constituted the elemental sulfur. Maintaining the hydration of this material at the stage of its formation is a deliberate procedure, as it facilitates its transport or possible further processing. The waste used was not contaminated with heavy metals (2.95 g Fe kg^−1^ d.m. and trace amounts of Cd, Cr, Cu, Mn, Ni, Pb and Zn). The content of nutrients in sulfur pulp was insignificant, considering agricultural use.

As the plasticity of sulfur pulp is negligible, granulation binders are required. Herein, as a support material stabilizing waste sulfur properties, halloysite (aluminosilicate clay mineral) was introduced. Its advantage is that it is inexpensive, easily available and occurs naturally. The halloysite used in this experiment was obtained from the “Dunino” open pit mine, located in Dunino (southwestern Poland). The halloysite grain agglomerates consisted of individual loosely spaced particles (crystals) in the form of nanotubes of various diameter and length, as well as of nanoplates (Figure 2). This material was naturally saturated with iron and titanium oxides (Figure 1e), e.g., magnetite, magnesioferrite, ilmenite, hematite and goethite. Iron oxides cause pigmentation of halloysite to a rusty-reddish color. In this study, dried halloysite powder with a moisture content of 5% was used.

In the experiment, Iber-Humus Ps-90 Ultra, a solid fertilizer produced from leonardite deposits, was used as a source of organic matter applied into the soil. According to the producers, this material contains 70% humic acids (HA), 15% fulvic acids, 8% K (in K_2_O form). It was characterized by pH 9.5, and fine-grained structure (Figure 1f).

### 2.2. Establishing the Incubation Experiment

The incubation experiment was set up on sandy soil, classified Hortic Cambisols Eutric [34]. The soil was collected from the area of southern Poland (50°6′48.3″ N 22°40′26.9″ E), from a 0–20 cm layer. This material was characterized by acid reaction, low content of sulfate sulfur and total sulfur, and was not contaminated with heavy metals (Table 1).

The experiment comprised five treatments (each treatment was conducted in triplicate): soil with no additions (control)—treatment C; soil with the addition of fertilizer A—treatment I; soil with the addition of fertilizer B—treatment II; soil with the addition of fertilizer C—treatment III; soil with the addition of fertilizer D—treatment IV.

The study included two series: the first one was unlimed (0Ca) and the second one was limed (+Ca). Calcium carbonate was introduced to the soil two months prior to adding the fertilizers.

The doses of fertilizers A, B, C and D were calculated so as to introduce the same amount of sulfur—20 mg S kg^−1^ d.m. of soil. Dose of humic acids amounted to 10 mg S kg^−1^ d.m. of soil (introduced with the fertilizers B and D).

The repetitions consisted of 280 g d.m. air-dried soil enriched with prepared fertilizers according to the experimental design. The soil was placed into plastic containers and was incubated at 25 ± 2 °C. Moisture content of the soil during the experiment was controlled and adjusted to 60% of its maximum water capacity. Water loss was restored every 3 days with deionized water. Soil samples were collected on the day of adding the fertilizers (immediately after application of the materials) as well as 15, 30, 60 and 90 days after their introduction, air-dried and sieved (1 mm mesh size) to prepare for analyses.

### 2.3. Methods of Laboratory Analyses

To characterize the properties of the prepared fertilizer granulates and raw components, the following analyses were conducted: structure of fertilizer components and prepared granulates was analyzed with an optical microscope Genetic Pro Bino (Delta Optical, Nowe Osiny, Poland); surface morphology of raw halloysite grains was analyzed with a scanning electron microscope S-3400N (Hitachi, Tokyo, Japan); moisture content of granulates was determined according to the requirements of the standard [35]; the samples were weighted with a scale with accuracy of 0.001 g and dried for 24 h at a temperature of 105 °C; moisture content of each granulate was calculated as a percentage from the Equation (1):

(1) $$MC=100-\left(m_{2}\times 100/m_{1}\right)$$

where: *MC*—moisture content (%); *m*_1_—mass of the sample before drying (g); *m*_2_—mass of the sample after drying (g).

Granulometric composition of granulates was determined according to the requirements of the standard [36]; using a set of sieves with the following mesh sizes: 0.25 mm, 0.5 mm, 1.02 mm, 2 mm, sample portion was sieve; after that, the remaining mass was weighed, and the percentages of the sample fraction of particles were calculated from the Equation (2):

(2) $$PS=m_{2}\times 100/m_{1}$$

where: *PS*—particle size (%); *m*_1_—mass of the test portion (g); *m*_2_—mass of the residue on the sieve (g).

Loose bulk density of fertilizer granulates was determined according to the requirements of the standard [37] by using an empty cylinder, determining the mass of the material in a known volume from the Equation (3):

(3) $$BD=m_{2}-m_{1}/V$$

where: *BD*—bulk density (kg m^−3^); *m*_1_—mass of the empty cylinder (kg); *m*_2_—mass of the cylinder including the material (kg); *V*—volume of the cylinder (m^3^).

To characterize the soil properties after introducing the prepared fertilizer granulates, pHKCl, electrolytic conductivity and sulfate content were determined. Soil pHKCl was determined potentiometrically in a 1 mol/L KCl suspension (Chempur, Piekary Śląskie, Poland) (1:2.5 *m*/*v*). To calculate the mean pH, pH values of replicates were converted into hydrogen ion [H+] concentrations, then the arithmetic mean was calculated and converted into pH according to the formula: pH = −log[H+]. Electrolytic conductivity (EC) was measured according to the requirements of the standard [38]; a soil:water (1:5 *m/v*) suspension was prepared using deionized water and mixed for 30 min at 40 rpm. Sulfate sulfur content was measured after extraction with three reagents of different extraction strengths:

- 0.03 mol L^−1^ CH_3_COOH (Chempur, Piekary Śląskie, Poland); the extraction was conducted at 1:10 (*m/v*) ratio, the suspensions were shaken for 30 min at 30 rpm, filtrated and analyzed by inductively coupled plasma-optical emission spectrometry (ICP-OES), on an Optima 7300 DV spectrometer (Perkin-Elmer, Waltham, MA, USA);
- unbuffered, neutral 0.01 mol L^−1^ CaCl_2_ (POCH, Gliwice, Poland); the extraction was conducted at 1:10 (*m/v*) ratio, the suspensions were shaken for 2 h at 40 rpm, filtrated and analyzed by ICP-OES method;
- Mehlich 3 (M3) (pH 2.5) (0.2 mol L^−1^ CH_3_COOH (Chempur, Piekary Śląskie, Poland), 0.25 mol L^−1^ NH_4_NO_3_ (Chempur, Piekary Śląskie, Poland), 0.015 mol L^−1^ NH_4_F (Chempur, Piekary Śląskie, Poland), 0.013 mol L^−1^ HNO_3_ (Chempur, Piekary Śląskie, Poland), 0.001 mol L^−1^ EDTA (POCH, Gliwice, Poland)); the extraction was conducted at 1:10 (*m/v*) ratio, the suspensions were shaken for 5 min at 40 rpm, filtrated and analyzed by ICP-OES method.

Samples collected on all incubation dates were extracted with 0.03 mol L^−1^ CH_3_COOH (samples collected on all dates were also subjected to pH and EC determination). Moreover, for more detailed characteristics of the soils, the samples collected on the day of adding the fertilizers and 90 days after their introduction were extracted with 0.01 mol L^−1^ CaCl_2_ and Mehlich 3.

To characterize the soil properties before establishing the experiment, additional analyses were performed. Granulometric composition was determined by the Bouyoucos-Casagrande’s areometric method in Prószyński’s modification. Soil pHH_2_O was measured potentiometrically, using deionized water, at 1:2.5 (*m*/*v*) ratio. To measure hydrolytic acidity, the samples were treated with 1 mol L^−1^ Ca(CH_3_COO)_2_ (EUROCHEM BGD, Tarnów, Poland) using 1:2.5 (*m*/*v*) ratio. The suspensions were shaken for 1 h, filtered and titrated with 0.1 mol L^−1^ NaOH. The hydrolytic acidity was calculated from the amount of base used. To measure the maximum water capacity, the difference in soil mass before and after moisture conditioning by capillary rise was determined. Total carbon and nitrogen concentrations were determined with an automatic vario MAX cube CNS analyzer (Elementar Analysensysteme GmbH, Langenselbold, Germany). To determine the total content of sulfur (S), the samples were dry mineralized using 2 mol L^−1^ Mg(NO_3_)_2_ (Merck KGaA, Darmstadt, Germany) (12 h, 450 °C), and then the remains were dissolved in 31% HNO_3_ (POCH, Gliwice, Poland). To determine the total content of other nutrients (Fe, Cu, Mn, Ni, Zn) and trace elements (Cd, Cr, Pb), the samples were dry mineralized (12 h, 450 °C) and digested in a mixture of concentrated HClO_4_ (EUROCHEM BGD, Tarnów, Poland) and HNO_3_ (POCH, Gliwice, Poland) at 2:3 (*v*/*v*) ratio. The content of elements in the obtained extracts was determined by ICP-OES method.

### 2.4. Statistical Analysis of the Results

The results obtained in the incubation experiment were elaborated statistically, providing the values of arithmetic mean and standard deviation (SD). The obtained results underwent a statistical analysis, by conducting a one-way analysis of variance for the system with repeated measures (qualitative factor: treatment; repeated measures factor: date of analysis). The significance of differences in mean values was assessed using the Tukey test, with the significance level *p* ≤ 0.05. Dell Statistica (data analysis software system), version 13 (Dell Inc., Tulsa, OH, USA) program was used.

The *t*-test was used for dependent samples in the analysis of variance, i.e., two variables were compared for the same set of observations. The variance was calculated as the sum of squares of deviations from the mean, divided by n-1 (sample size minus one). Therefore, at given n, the variance is a function of the sum of squares (deviations from the mean). The F-test is largely resistant to deviations from the norm. During the analyses discussed, the authors observed the size of kurtosis. If it was greater than 0, then the F value tends to be low, and the null hypothesis could not be rejected, even if it is not true. The opposite occurs when the value of kurtosis is less than 0. The skewness of the distribution usually does not have a significant impact on the value of the F statistic. If the counts per cell are large enough, then deviations from the normal distribution do not matter because of the central limit theorem, which establishes that the sample means follow a normal distribution, regardless of the distribution of the variable in the population.

## 3. Results and Discussion

### 3.1. Physical Properties of Fertilizer Granules

**Figure 3:**
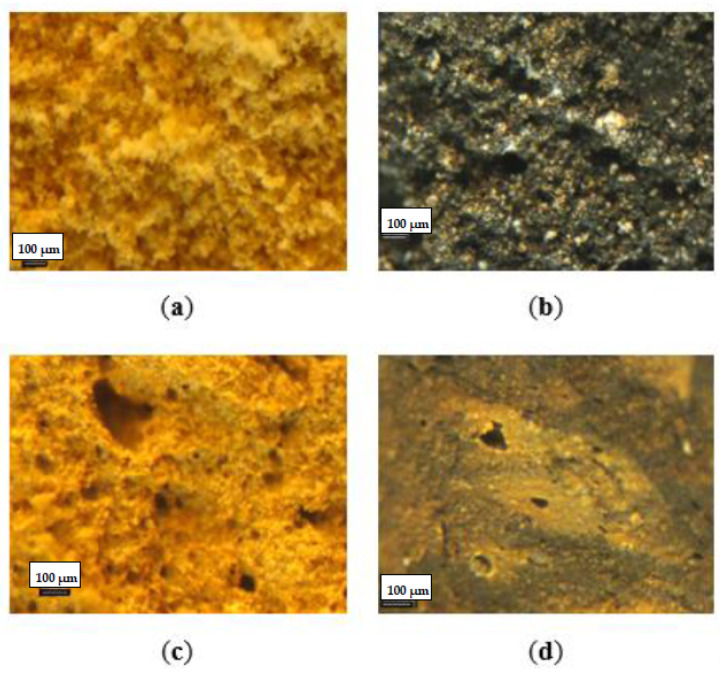
Optical microscope view of fertilizer granule structure of fertilizer A (**a**), fertilizer B (**b**), fertilizer C (**c**), fertilizer D (**d**).

The granules of the prepared fertilizers had a porous structure (Figure 3a–d). Granules of fertilizers A, B and C were characterized by smaller compaction than granules of fertilizer D, whose structure was visibly more concentrated.

The moisture content of fertilizer granulates ranged from 0.822 to 2.088% (Table 2). Fertilizers A and D had the lowest and highest water content, respectively. The moisture content of fertilizers A and C as well as B and D showed similar values.

The hygroscopic nature of fertilizer, and thus its ability to absorb moisture from the atmosphere, may be problematic during its handling and application [39]. Several factors, particularly moisture content, affect the formation of agglomerates (caking) during fertilizer storage. Generally, most fertilizers have a tendency towards caking, resulting from the growth of crystal bonds between fertilizer granules, and it can be observed over weeks or months in storage. To ensure fertilizer non-caking, the critical moisture content of the final product should not exceed 0.5% for mineral fertilizers containing nitrogen in the form of NO_3_^−^, NH_4_^+^ and NH_2_, <1.0% and <1.5% for mineral fertilizers with N:P_2_O_5_ ratio of 1:1 or higher or less than 1:1, respectively. Critical moisture for fertilizers without ammonium nitrate or urea and for superphosphates is <2.0 and <2.5, respectively. To keep the fertilizer in good physical condition, conditioners are applied. However, this treatment is not essential with all fertilizers. If possible, other means are recommended to avoid caking, e.g., proper drying [40].

Based on the sieve analysis, the grain composition of individual fertilizer granulates was determined, constituting the percentage share of granulometric fractions in relation to the total amount of sieved material. Fertilizer A was characterized by the highest fragmentation, the grain fraction <0.25 mm constituted 48.3% of the total fertilizer amount (Table 3). Fertilizers B, C and D were characterized by significantly lower fragmentation, with the sum of the percentage share of grains with a diameter ranging from 0.50 to >2.0 mm constituting 81.8%, 88.4% and 74.6% (by weight), respectively (Table 2).

In general, because of practical and safety considerations, elemental sulfur is not applied to soil in powdered form, but as granules, pastilles or prills. The effectiveness of granulation is affected, among other things, by the wettability of the components. Waste sulfur is a material with hydrophobic properties. The large share of this material in fertilizer mixtures leads to the formation of weak, fragile granules with high percentage of fines. Weakness of granules and their high tendency to degrade to fines result in dustiness, which is undesirable. This property is important especially in bulk handling and spreading. When considering the production of fertilizer based on predominant share of waste sulfur on a larger scale, granulation parameters need to be adjusted to increase the susceptibility of the sulfur powder to compaction, as well as its ability to retain the shape obtained during granulation. Increased halloysite addition and inclusion of humic acids into the mixtures led to the formation of larger diameter granules. Sulfur filled the pores of the other components; thus, the granule structure was more stable.

The size distribution of granules can have several impacts on physical properties of prepared fertilizers. For example, it takes granules with larger diameter more time to dissolve in water than granules with smaller size. Fertilizers in powdered or finely divided form readily cake during storage. Fertilizer particle size and size distribution have a direct effect on spread width and uniformity. Granules with diameters ranging from 1 to 5 mm are most suitable for uniform mechanical application using field equipment. As they tend to produce less dust, the product losses (e.g., caused by the wind) and concurrent environmental problems are reduced [41].

Based on the bulk density value, constituting the mass per unit volume of material in loose state, fertilizer A was classified as a light material with the lowest value of bulk density of 376.6 kg m^−3^ (Table 2). Fertilizers B, C and D were classified as medium materials, with bulk density value ranging of 0.6–0.9 Mg m^−3^. Fertilizer D had the highest value of bulk density. The increase in bulk density of particles is related to their increasing cohesiveness.

Product manufacturing technologies should take into account the quality requirements related to resistance to environmental factors [42,43]. The most common method of mechanical stabilization of fertilizers is granulation. Granulation, referred to as agglomeration or pelletizing, is one of the ways of material densification. It is a mechanical process taking place under specific moisture, heat, and pressure conditions [44,45]. The densification of small material particles into larger granules increases the bulk density, and thus may reduce the costs and difficulties regarding the process, transport, storage, and utilization of materials characterized by low value of bulk density. Thus, density properties are important as they translate into the volume necessary to store, transport, and handle, as well as into calibration of fertilizer spreading equipment [39,46]. Bulk density values for granular material range from close to 400 kg m^−3^ for lighter materials [47], and close to 1900 kg m^−3^ for heavier materials [48]. This parameter depends on the particle arrangement, and is a function of grain size and shape, and grain size distribution [49]. Klinkmüller et al. [39] concluded that, during sieving, rounded grains form a closer packing than angular particles. Taking into account equal grain density and size distribution, rounded particles are characterized by a higher bulk density, resulting from closer packing.

Halloysite, similar to humic substances, exhibits a hydrophilic nature. The addition of this material in varied amounts to mixtures explains the increase in moisture content of prepared fertilizers. Components of the mixtures filled the micropores of the halloysite grain, and thus an increase in grain size and bulk density of the prepared fertilizers has been observed.

### 3.2. Soil properties after Fertilizer Application

The pH of the soil solution is an indication of the hydrogen ion [H+] activity. This parameter is an important indicator of soil health, shaping its properties [50].

During the incubation experiment, the pH of the unlimed soil and the limed soil ranged from 4.56 to 4.81 and from 5.21 to 5.48, respectively (Table 3); both ranges indicate acid reaction. Regardless of the set of treatments—limed or unlimed—the influence of the used fertilizers on soil pH was small, as were the changes in pH during the experiment. However, application of fertilizers with the largest proportion of halloysite and humic acids (fertilizers B, C and D) stabilized the soil pH throughout the experiment (compared to fertilizer A, containing only a small dose of halloysite).

Świercz et al. [41], in a pot experiment, reported that halloysite addition to the soil at the rate of 10, 30 and 50% increased its pH. In addition, Radziemska [21], in a pot experiment, found that 3% of halloysite addition caused a significant increase in pH, whereas Belal et al. [44], in their field experiment, found that soil pH decreased gradually with increasing application rates of HA (0, 50, or 100 kg ha^−1^) and elemental sulfur (0, 200, or 400 kg ha^−1^) individually or in integration (but in this case no Dunino halloysite was used). Furthermore, a minimum soil pH reduction through the integrative application was obtained by adding 50 kg ha^−1^ of HA and 200 kg ha^−1^ of elemental sulfur. The authors pointed out that sulfuric acid production under microbial oxidation of elemental sulfur, combined with the acidity of HA, leads to a reduced value of soil pH.

Soil acidification occurs naturally. However, this process can be also accelerated by intensive crop production or prevented by sustainable management practices. A decrease in soil pH, caused by acidification, has a harmful effect on nutrient cycles, soil structure, biodiversity of biota, and agricultural productivity [51,52,53].

Elemental sulfur oxidation induces formation of H_2_SO_4_ in soil, and hence its acidification. In addition, H_2_SO_4_ reacts with the native soil CaCO_3_, and CaSO_4_ is created. The created CaSO_4_ can be ionized to Ca^2+^ and SO_4_^2−^, which leads to further reduction of soil pH. However, the degree of acidification varies depending on the amount of elemental sulfur applied and on the soil buffering capacity [54]. Zhao et al. [48] reported soil acidification induced by elemental sulfur application. Similarly, Karimizarchi et al. [49] found a decrease in soil pH after 40 days of incubation with different doses of elemental sulfur (0, 0.5, 1 and 2 g S kg^−1^ of soil). A decrease in pH is also expected to occur at lower elemental sulfur doses used in the field due to repeated application. Bobowiec and Tabak [50], under 150 days of incubation, found that fertilization with elemental sulfur at doses of 10, 20, 30, 60 mg S kg^−1^ caused a decrease in soil pH. At the same time, the authors highlighted that soil liming reduced soil acidification. While Ye et al. [51], in a field experiment with soil fertilized with elemental sulfur at rates of 0, 112, 224, and 448 kg S ha^−1^, did not find any significant effect of this treatment on soil pH. As the authors pointed out, the limited effect of acidification may result from the elemental sulfur application rate too low to cause the decrease in pH, and from the high buffering capacity of treated soil. However, it is recommended to monitor soil pH after application of elemental sulfur-containing fertilizer to correct soil acidification by liming, if necessary. Liming is an essential treatment for good soil management, plant growth and nutrient use efficiency.

The value of electrical conductivity determines the level of soil salinity, which is expressed as concentration of soluble salts in soil water. Soil is classified as saline while the EC of saturation extract in the root zone exceeds 4 mS cm^−1^ at 25 °C [55]. However, plant growth and development may be disturbed or inhibited under lower EC. At a relatively low salinity level, a critical value of EC for crop production varies over time for the same crop and site, since plant yield is affected by various factors [56]. It has been assessed that 20% of cropland and 33% of irrigated land is affected by salinity and degraded worldwide. Furthermore, due to various reasons, including natural and anthropogenic factors, this area is constantly increasing [57,58]. Salinity causes dispersion of soil particles, which is accompanied by increasing soil compactness. The damage of soil structure entails reduction of oxygen availability, infiltrability and hydraulic conductivity in the root zone. Due to the high concentration of sodium, soil pH increase. It may further lead to soil alkalization [59]. The excess of dissolved salts suppresses the growth of plants [60]. Crops cultivated on saline soils suffer due to nutritional disorders and toxicities, poor soil conditions and osmotic stress that limit water uptake (physiological drought) [61]. All soils naturally contain soluble salts. However, unsuitable management of agriculture practices (fertilization, irrigation) may induce soil salinity [62,63,64,65].

For both sets of treatments—limed and unlimed—the value of soil electrolytic conductivity was rather stable for the first 30 days of the incubation experiment (Table 4). The highest EC was recorded in the 60th day of incubation. In the successive period (i.e., after the 90-day experiment), the soil EC decreased, but not to the level recorded at the beginning of the experiment. At the end of incubation, no statistically significant effect of the examined treatments on the soil EC was found.

The results presented in this study do not coincide with those presented by Pourbabaee et al., [59] and Yang et al. [60], who highlighted the influence of elemental sulfur fertilization on soil EC. However, the effect of material application on EC may depend on this material dose. Yang et al. [60] investigated the effect of introduction of 0.15 g elemental sulfur into 10 g of soil. After 12 weeks of incubation experiment, the authors found an increase in soil conductivity from 0.19 to over 2.0 mS cm^−1^. The increase in electrical conductivity can be explained by microbiological oxidation of elemental sulfur, which is accompanied by sulfuric acid production. The dissolution of CaCO_3_ by H_2_SO_4_ is revealed as increased accumulation of soluble salts, which can be measured by electrical conductivity [66]. Gümüş and Şeker [62], after performing a 62-day soil incubation, concluded that EC increased linearly in response to incrementing doses of humic acids (0, 0.5, 1, 2 and 4%). The application of humic acids may be burdened by an increase in soil EC due to rich nutrient composition of this material [67]. Applying conventional mineral fertilizers that contain nitrogen can also increase the content of easily soluble salts in the soil [68]. Other results are presented by Belal et al. [44], who under a field experiment found that EC of soil decreased gradually with increasing application rates of HA (0, 50, or 100 kg ha^−1^) and elemental sulfur (0, 200, or 400 kg ha^−1^) individually or in integration. Maximum soil EC reduction (48.3%), through the integrative application, was obtained by addition of 100 kg ha^−1^ of HA and 400 kg ha^−1^ of elemental sulfur. Halloysite “Dunino” has a large specific surface area (65–75 m^2^ g^−1^) and large porosity (65–75%), and it can intercalate a wide range of different salts and organic compounds in its spatial structure [69].

According to literature [70,71,72,73], halloysite is characterized by a wide range of cation exchange capacity, and in some forms of this mineral, values of up to 50 cmol(+) kg^−1^ were reported. High temperature dehydration of halloysite contributes to losses of intercalate capability, and adsorption of macromolecules generally occurs through loading into the inner tube lumens [74].

Throughout the growing season, crops need to take up sufficient amounts of sulfur for optimum quality and quantity of yield (sulfur is the fourth primary plant nutrient) [75]. Due to the constantly increasing sulfur deficiency caused by intensive farming practices and its decreased deposition from the atmosphere, increased consumption for sulfur fertilizers has been observed, meeting short- and long-term crop requirements [76]. There is a wide variety of fertilizers, containing sulfur in the form of sulfates, elemental sulfur or thiosulfates [77]. The use efficiency of sulfur fertilizers is low, in some cases it is below 10% [78,79]. Transformation and fate of applied fertilizer is shaped by its chemical form, solubility in soil, interaction with minerals, pH, organic matter, and the presence of other anions. There is a growing demand for elemental sulfur fertilizers due to high concentration of nutrient, negligible leaching losses and residual effect observed in subsequent growing seasons, and also due to economic aspects since elemental sulfur application and transport costs are low [80,81].

Sulfur directly available to plants occurs in the form of inorganic sulfate ions SO_4_^2−^ dissolved in the soil solution [82]. Nonetheless, this fraction is not the only one that can be taken up by plants. Sulfates adsorbed on soil particles, bound by weak binds, can be released into the soil solution and further absorbed by plant roots. Thus, these ions are also considered as bioavailable [83]. Elemental sulfur is not directly available for plants, until it is oxidized to sulfate ions SO_4_^2−^ [20]. There are many factors that influence the oxidation process. Soil pH ranging from 5.4 to 8.0, higher content of organic matter, soil moisture content close to field capacity and temperatures varying between 30 and 40 °C [84,85,86,87,88], maximize the rate of elemental sulfur oxidation. Sulfur deficiency decreases the plant yield, whilst a sufficient supply of this nutrient increases plant efficiency, including nitrogen utilization and protein synthesis [51,89]. Rego et al. [83] are of the opinion that there is a necessity to supply this element if the content of soil SO_4_^2−^ ions is below 10 mg S g^−1^.

The strength of the reagents used for the extraction of sulfate sulfur grew increased in the following order (Table 5): 0.03 mol L^−1^ CH_3_COOH < 0.01 mol L^−1^ CaCl_2_ < Mehlich 3. Application of all the examined fertilizers increased sulfate sulfur content in the soil significantly (compared to the control soil with no additions). However, the effect of individual fertilizers on the sulfate sulfur content in the soil was small, which can be explained by the same sulfur dose (20 mg S kg^−1^ d.m.) added with individual fertilizers. After 90 days of incubation, differences in sulfate sulfur content between the fertilized treatments were visible only after the use of strong extractants (0.01 mol L^−1^ CaCl_2_ and Mehlich 3); the highest sulfate sulfur content for both sets of treatments—limed and unlimed—was recorded in the soil with the addition of fertilizers B (waste sulfur (81%) + halloysite (5%) + humic acids (14%) and C (waste sulfur (50%) + halloysite (50%).

The obtained results correspond with our previous findings regarding the suitability of waste sulfur as a source of sulfate sulfur in soil, and the beneficial effect of liming on the oxidation of this material [57]. The efficiency of adding elemental sulfur to soil has also been reported by other authors. Ye et al. [51] found that after elemental sulfur application the content of sulfates in the soil increased. While Mattiello et al. [15] tested the effect of co-granulated elemental sulfur with bentonite clay and found that this treatment increased the content of sulfates in incubated soils with contrasting pH. Authors concluded that the rate of elemental sulfur oxidation was greater in the acidic than in the slightly alkaline soil.

### 3.3. Comparison of Soil Extractants

Nutrients occur in soil in water-soluble, exchangeable, and non-exchangeable fractions. Their availability to plants depends on the dynamic equilibrium among different fractions. There are various extraction methods that reflect the level of element availability [58,90].

Extraction with CH_3_COOH gives information about the potentially bioavailable form of sulfur [91]. Nutrient extraction using 0.01 mol L^−1^ CaCl_2_ solution represents soluble SO_4_^2−^ and dissolved organic sulfur. Mehlich 3 reflects the content of soluble SO_4_^2−^ with an increased share of organic and adsorbed sulfur. CaCl_2_ solution is a commonly used method for sulfate extraction and is favored for the extraction of slightly acidic to neutral soils [92]. Cl-based extraction solutions mobilize water-soluble SO_4_^2−^. Due to the formation of poorly soluble CaSO_4_, CaCl_2_ solution is characterized by a relatively low extraction power. Mehlich 3, as more acidic, extracts soluble, exchangeable, and some of non-exchangeable compounds [93]. By using this method, it is possible to easily and quickly determine the content of bioavailable forms of elements. This solution is also recommended to extract sulfur, as it consists of acetate and nitrate anions capable to replacing adsorbed SO_4_^2−^, and is suitable for acidic to neutral soils [94,95,96,97,98].

In the conducted research, lower sulfur content was determined for the CaCl_2_ method, which is consistent with the findings reported by Ketterings et al. [85]. The higher amounts of sulfur extracted with Mehlich 3 were expected, due to the fact that Mehlich 3 is one of the strongest extractants to determine bioavailable fractions of nutrients. Weak salt solutions are not efficient in extraction of adsorbed SO_4_^2−^ [86,87]. Kulhánek et al. [99], studied a set of 98 samples of agricultural soils used in various ways and concluded that sulfur content determined by Mehlich 3 extractant closely corresponds with the amounts of bioavailable fraction of this element.

## 4. Conclusions

One of the strategic directions for the development of the fertilizer industry is the use of waste materials for the production of fertilizers. Using additions that improve the properties and increase the effectiveness of waste-based fertilizers corresponds with the modern approach to creating waste-free technologies, observed in agriculture and other branches of the economy. Waste sulfur has a high fertilizing potential due to the high content of this element. The problem of using this waste for fertilization purposes is its physical properties. These properties make it impossible to granulate this material and make the application much more difficult. The results of the presented research show that the addition of hallosite and humic extract allows to improve the properties of the fertilizer both from the point of view of granulation technology and from the point of view of influencing soil properties. So far, no one has used such materials for the technological valorization of waste sulfur. We are the first to show that enrichment of the waste sulfur-based fertilizers with humic acids and halloysite improves the fertilizer properties—especially the share of larger granules and bulk density. Moreover, the prepared fertilizers are a valuable source of sulfur, which may become available to plants. However, this manuscript describes a preliminary study. To confirm, further research into the proposed fertilizers is required.

## Figures and Tables

**Table 1:** Soil properties before establishing the experiment.

Parameter	Value
Soil texture, %	
Fraction sand 2–0.05 mm	88
Fraction dust 0.05–0.002 mm	10
Fraction loam < 0.002 mm	2
Maximum water capacity, %	20.55
pH_H2O_	6.01
pH_KCl_	5.04
Hydrolityc acidity, mmol (+) kg^–1^ d.m.	14.93
Electrical conductivity, μS cm^–1^ ± SD	91
Sulfate S—extraction with 0.03 mol L^−1^ CH_3_COOH, mg kg^–1^ d.m	3.57
Sulfate S—extraction with 0.01 mol L^−1^ CaCl_2_, mg kg^–1^ d.m	3.86
Sulfate S—extraction with Mehlich 3, mg kg^–1^ d.m	22.10
Total N, g kg^–1^ d.m.	0.61
Total C, g kg^–1^ d.m.	8.23
Total S, mg kg^−1^ d.m.	97.5
Total Fe, g kg^−1^ d.m.	2.70
Total Cd, mg kg^−1^ d.m.	0.683
Total Cr, mg kg^−1^ d.m.	3.86
Total Cu, mg kg^−1^ d.m.	4.27
Total Mn, mg kg^−1^ d.m.	106
Total Ni, mg kg^−1^ d.m.	3.18
Total Pb, mg kg^−1^ d.m.	7.13
Total Zn, mg kg^−1^ d.m.	23.9

**Table 2:** Properties of fertilizer granulates.

		Percentage Share of Particular Fractions of Particle Diameters in mm ± SD					
Fertilizer	Moisture, % ± SD	<0.25	0.25–0.50	0.50–1.02	1.02–2.0	>2.0	Bulk Density, kg m^−3^ ± SD
A	0.822 ± 0.027	48.2 ± 0.9	9.55 ± 0.27	11.8 ± 0.3	20.4 ± 0.1	9.84 ± 0.22	376 ± 9.77
B	1.78 ± 0.05	8.26 ± 0.02	9.97 ± 0.05	19.7 ± 0.5	34.7 ± 0.8	27.2 ± 0.3	626 ± 2.18
C	0.904 ± 0.020	5.26 ± 0.15	6.34 ± 0.11	15.9 ± 0.1	33.4 ± 0.2	39.0 ± 0.3	759 ± 18.3
D	2.08 ± 0.08	13.7 ± 0.3	11.6 ± 0.2	21.5 ± 0.6	30.4 ± 0.3	22.5 ± 0.5	917 ± 13.2

SD—standard deviation.

**Table 3:** pH_KCl_ of soil during the experiment.

Treatment		Incubation Time (Days)				
		0	15	30	60	90
0Ca	C ^1^	4.70 abc ^2^	4.71 bc	4.68 abc	4.61 ab	4.57 ab
	I	4.71 bc	4.81 c	4.61 ab	4.63 ab	4.56 a
	II	4.72 bc	4.65 abc	4.66 abc	4.62 ab	4.58 ab
	III	4.72 bc	4.66 abc	4.63 abc	4.65 abc	4.69 abc
	IV	4.71 bc	4.71 bc	4.65 abc	4.69 abc	4.72 bc
+Ca	C	5.40 efgh	5.28 abc	5.29 abcd	5.39 defgh	5.41 fgh
	I	5.36 bcdefgh	5.30 bcde	5.21 a	5.31 bcdef	5.30 bcde
	II	5.37 cdefgh	5.35 bcdefg	5.30 bcdef	5.46 gh	5.33 bcdef
	III	5.36 cdefgh	5.26 ab	5.27 ab	5.44 gh	5.35 bcdefg
	IV	5.41 fgh	5.35 bcdefg	5.31 bcdef	5.48 h	5.34 bcdefg

^1^ C: soil with no additions (control); I: soil with the addition of fertilizer A (waste sulfur (95%) + halloysite (5%)); II: soil with the addition of fertilizer B (waste sulfur (81%) + halloysite (5%) + humic acids (14%)); III: soil with the addition of fertilizer C (waste sulfur (50%) + halloysite (50%)); IV: soil with the addition of fertilizer D (waste sulfur (46%) + halloysite (46%) + humic acids (8%)). ^2^ for a given set of treatments—limed or unlimed—mean values marked with the same letters do not differ statistically significantly at the significance level *p* ≤ 0.05; according to Tukey test. Different letters mean statistically significant differences at the significance level *p* = 0.05.

**Table 4:** Electrical conductivity of soil during the experiment (μS cm^−1^ ± SD).

Treatment		Incubation Time (Days)				
		0	15	30	60	90
0Ca	C ^1^	129 a ^2^ ± 8	135 a ± 1	139 a ± 12	245 gh ± 6	179 abcdef ± 13
	I	144 ab ± 9	153 abc ± 7	142 ab ± 18	256 h ± 41	206 cdefgh ± 8
	II	139 a ± 13	155 abc ± 7	141 ab ± 15	231 fgh ± 39	195 bcdefg ± 11
	III	133 a ± 10	143 ab ± 1	148 ab ± 14	176 abcde ± 3	222 efgh ± 8
	IV	149 ab ± 13	166 abcd ± 3	172 abcde ± 26	219 defgh ± 34	218 defgh ± 12
+Ca	C	138 ab ± 6	131 ab ± 2	142 abc ± 15	225 gh ± 20	179 abcdefg ± 7
	I	136 ab ± 9	143 abc ± 2	143 abc ± 24	180 bcdefg ± 23	200 efgh ± 1
	II	134 ab ± 18	138 ab ± 1	140 ab ± 9	177 abcdefg ± 19	195 defg ± 5
	III	129 a ± 13	150 abcde ± 11	157 abcdef ± 17	277 i ± 33	192 cdefg ± 14
	IV	145 abcd ± 4	154 abcdef ± 4	203 fgh ± 26	250 hi ± 33	192 cdefg ± 7

For SD, see Table 2. ^1,2^ see Table 3. Different letters mean statistically significant differences at the significance level *p* = 0.05.

**Table 5:** Sulfate sulfur content in soil during the experiment (mg S-SO_4_^2–^ kg^−1^ d.m. ± SD).

		Extraction with 0.03 mol L^−1^ CH_3_COOH					
Treatment		Incubation Time (Days)					
		0	15	30		60	90
0Ca	C ^1^	4.58 a ^2^ ± 0.67	7.78 abc ± 0.21	12.73 cd ± 0.55		6.85 abc ± 0.57	11.05 bc ± 0.25
	I	6.68 ab ± 6.68	23.68 fg ± 2.32	34.59 i ± 3.29		27.92 gh ± 3.79	17.03 de ± 1.68
	II	7.13 abc ± 7.13	21.32 ef ± 0.94	31.30 hi ± 2.48		31.37 hi ± 2.42	18.53 ef ± 1.79
	III	7.85 abc ± 0.89	20.79 ef ± 0.28	32.79 hi ± 2.82		35.18 i ± 3.23	19.09 ef ± 0.96
	IV	7.22 abc ± 0.42	19.93 ef ± 0.84	32.67 hi ± 1.28		30.63 hi ± 2.42	18.00 de ± 0.37
+Ca	C	5.19 ab ± 0.49	9.53 abcd ± 0.60	12.40 bcde ± 0.49		11.79 bcde ± 0.84	2.97 a ± 0.40
	I	12.06 bcde ± 0.69	24.92 fgh ± 1.64	41.59 j ± 6.56		41.67 j ± 2.02	15.50 cde ± 0.94
	II	8.23 abc ± 0.44	14.13 bcde ± 0.29	20.40 efg ± 0.77		33.72 hij ± 0.83	15.88 cde ± 0.77
	III	11.02 abcd ± 0.83	17.69 def ± 1.56	32.47 hi ± 5.52		34.59 ij± 5.03	12.05 bcde ± 0.37
	IV	11.77 abcde ± 0.53	16.38 cdef ± 2.15	28.90 ghi ± 7.58		33.52 hij ± 4.26	9.93 abcd ± 1.60
Extraction with 0.01 mol L^−1^ CaCl_2_				Extraction with Mehlich 3			
Treatment		Incubation time (days)		Treatment		Incubation time (days)	
		0	90			0	90
0Ca	C ^1^	7.95 a ± 1.02	10.41 a ± 1.15	0Ca	C ^1^	23.55 a ± 1.60	28.49 a ± 0.17
	I	11.72 a ± 0.12	32.07 b ± 3.30		I	33.04 a ± 3.98	56.32 b ± 3.42
	II	13.08 a ± 0.30	35.26 bc ± 2.82		II	28.58 a ± 0.45	68.58 c ± 6.58
	III	11.03 a ± 1.10	40.05 c ± 1.78		III	29.18 a ± 2.44	71.74 c ± 1.28
	IV	13.31 a ± 2.22	32.97 b ± 2.64		IV	31.50 a ± 3.40	62.60 bc ± 7.64
+Ca	C	7.76 a ± 0.65	11.97 a ± 1.72	+Ca	C	28.20 ab ± 0.53	30.77 ab ± 2.81
	I	14.03 a ± 0.39	40.39 c ± 1.54		I	35.98 b ± 1.79	77.29 d ± 2.76
	II	12.69 a ± 0.58	36.61 c ± 4.98		II	29.72 ab ± 2.21	76.05 d ± 4.45
	III	11.00 a ± 1.30	35.40 bc ± 2.61		III	26.60 ab ± 0.92	85.38 d ± 5.09
	IV	11.97 a ± 0.76	28.80 b ± 3.69		IV	22.16 a ± 0.49	61.69 c ± 6.57

SD see Table 2. ^1^ see Table 3. ^2^ for a given extractant and set of treatments—limed or unlimed—mean values marked with the same letters do not differ statistically significantly at the significance level *p* ≤ 0.05; according to Tukey test. Different letters mean statistically significant differences at the significance level *p* = 0.05.

## Data Availability

Not applicable.
